# Differences in the reliance on cuticular hydrocarbons as sexual signaling and species discrimination cues in parasitoid wasps

**DOI:** 10.1186/s12983-018-0263-z

**Published:** 2018-05-10

**Authors:** Jan Buellesbach, Sebastian G. Vetter, Thomas Schmitt

**Affiliations:** 1 Department of Science, Policy, & Management, University of California, 130 Mulford Hall, Berkeley, CA 94720-3114 USA; 2 Research Institute of Wildlife Ecology, Department of Integrative Biology and Evolution, University of Veterinary Medicine Vienna, Savoyenstr. 1, A-1160 Vienna, Austria; 3 Department of Animal Ecology and Tropical Biology, University of Würzburg, Am Hubland, D-97074 Würzburg, Germany; 4 Department of Evolutionary Biology and Animal Ecology, Faculty of Biology, University of Freiburg, Hauptstr. 1, D-79104 Freiburg, Germany; 5 Spemann Graduate School of Biology and Medicine (SGBM), Albert Ludwigs University Freiburg, Albertstr. 19 A, D-79104 Freiburg, Germany

**Keywords:** Chemical communication, Assortative mating, Mate recognition, Prezygotic reproductive isolation, Speciation, *Nasonia*, *Trichomalopsis*, *Muscidifurax*, Pteromalidae, Hymenoptera

## Abstract

**Background:**

Cuticular hydrocarbons (CHC) have been documented to play crucial roles as species- and sex-specific cues in the chemical communication systems of a wide variety of insects. However, whether they are sufficient by themselves as the sole cue triggering sexual behavior as well as preference of con- over heterospecific mating partners is rarely assessed. We conducted behavioral assays in three representative species of parasitoid wasps (Hymenoptera: Pteromalidae) to determine their reliance on CHC as species-specific sexual signaling cues.

**Results:**

We found a surprising degree of either unspecific or insufficient sexual signaling when CHC are singled out as recognition cues. Most strikingly, the cosmopolitan species *Nasonia vitripennis*, expected to experience enhanced selection pressure to discriminate against other co-occurring parasitoids, did not discriminate against CHC of a partially sympatric species from another genus, *Trichomalopsis sarcophagae*. Focusing on the latter species, in turn, it became apparent that CHC are even insufficient as the sole cue triggering conspecific sexual behavior, hinting at the requirement of additional, synergistic sexual cues particularly important in this species. Finally, in the phylogenetically and chemically most divergent species *Muscidifurax uniraptor,* we intriguingly found both CHC-based sexual signaling as well as species discrimination behavior intact although this species is naturally parthenogenetic with sexual reproduction only occurring under laboratory conditions.

**Conclusions:**

Our findings implicate a discrepancy in the reliance on and specificity of CHC as sexual cues in our tested parasitioid wasps. CHC profiles were not sufficient for unambiguous discrimination and preference behavior, as demonstrated by clear cross-attraction between some of our tested wasp genera. Moreover, we could show that only in *T. sarcophagae*, additional behavioral cues need to be present for triggering natural mating behavior, hinting at an interesting shift in signaling hierarchy in this particular species. This demonstrates the importance of integrating multiple, potentially complementary signaling modalities in future studies for a better understanding of their individual contributions to natural sexual communication behavior.

**Electronic supplementary material:**

The online version of this article (10.1186/s12983-018-0263-z) contains supplementary material, which is available to authorized users.

## Background

The reliance on sexual cues and signals for successful reproduction has been documented in a wide variety of animal species [1]. Any cues capable of accurately conveying information about species, sex, and reproductive status have the potential to contribute to finding, attracting, securing, and eventually reproducing with suitable mates [2, 3]. For closely related species occurring in sympatry, sexual signals with high degrees of similarity bear the risk of cross-attraction, which might in turn lead to fitness reductions due to heterospecific attraction, courtship, and mating. This has led to the assumption of increased diverging selection acting on sexual cues and signals and their corresponding recognition mechanisms in sympatric species, reducing the risk of cross-attraction [4, 5] and contributing to prezygotic reproductive isolation [6–9].

Insects, the most diverse animal class [10], have been documented to utilize chemical signaling as their predominant mode of communication [11, 12]. Cuticular hydrocarbons (CHC), the dominant fraction of the waxy lipid layer located on the insects’ epicuticle, constitute a particularly versatile group of semiochemicals being involved in a wide variety of signaling systems. Most prominently, CHC have been documented to play pivotal roles in sexual communication as sex attractants [13–15], and species recognition cues [16–18]. For instance, in closely related species of the Coleopteran families Cerambycidae [19], Lampyridae [20] and Chrysomelidae [21–23], preference of con- over heterospecific mating partners appears to be mediated by species-specific CHC differences. Similarly, crosses between the Lepidopteran species *Danaus erippus* and *Danaus plexippus* hint at CHC-mediated prezygotic reproductive isolation [24]. CHC involvement in species recognition and sexual signaling has further been demonstrated in the Hymenopteran wasp families Bethylidae [25] and Braconidae [26].

However, most studies on CHC-based sexual signaling mechanisms have been conducted in the fruit fly genus *Drosophila* [27–29]. For different *Drosophila* species, CHC profiles appear to be either sexually monomorphic or dimorphic [30]. In dimorphic species like *D. melanogaster*, qualitative and quantitative differences between males and females clearly separate their CHC profiles [30, 31]. It has been demonstrated that this distinction between sexually mono- and dimorphic CHC profiles contributes to the prevention of cross-attraction between these species [32–34]. Furthermore, it has been demonstrated between different *Drosophila* species that CHC-mediated mate discrimination and species recognition appear to be more pronounced in sympatric than in allopatric species [35–37]. These examples strongly suggest a distinctive role for CHC-based sexual signaling in prezygotic reproductive isolation between different *Drosophila* species. However, expanding the knowledge gained from *Drosophila* to other insect taxa will be indispensable for achieving a more holistic view on the various aspects of CHC-based sexual communication and its influence on reproductive isolation between sympatric species [38].

Recent advances have been made in deciphering CHC-based sexual signaling mechanisms in the jewel wasp species complex *Nasonia* (Hymenoptera: Pteromalidae), a well-established insect model system providing a unique set of features such as haplodiploidy, cross-fertility of its four species and sequenced genomes [39–41]. Concerning CHC signaling in *Nasonia*, it has been shown that female CHC extracts constitute sexual cues capable of eliciting male courtship behaviour in its most prominent and cosmopolitan member, *N. vitripennis* [42]. Expanding these findings to the whole *Nasonia* genus and assessing the species-specificity of female CHC cues, we recently discovered that CHC are distinctive enough to unambiguously characterize sexes and species [43], although the sexual signaling function in female CHC could not be unambiguously demonstrated for all *Nasonia* species [43, 44].

The present study attempts to assess the role of CHC in sexual signaling, their species-specificity and their potential as prezygotic hybridization barriers in a larger phylogenetic context. *Nasonia vitripennis* was tested together with two other genera of the parasitoid wasp family Pteromalidae, assessing whether CHC are sufficient as sexual signaling cues for triggering male courtship and copulation behavior, which is only rarely assessed in parallel in CHC signaling studies. Furthermore, we investigated whether males can discriminate con- from heterospecific females based on their CHC profiles, thus elucidating the degree of species-specificity in CHC-mediated sexual signaling to assess their potential function in prezygotic reproductive isolation.

The representative species of *Nasonia* and its two most closely related genera *Trichomalopsis* and *Muscidifurax* [45] chosen for this study were the cosmopolitan *N. vitripennis*, the wide-spread biological pest control agent *T. sarcophagae* [46, 47] and the naturally parthenogenetic *M*. *uniraptor* [48, 49], which regains the capability of producing males under laboratory conditions [50]. These unique and ecologically divergent genera were chosen to represent a broad variety within the Pteromalidae, one of the largest and economically most important families of parasitoid wasps [51, 52].

We hypothesized that pronounced differences in female CHC profiles and corresponding male perception capabilities would be apparent in genus-specific recognition and discrimination behavior, potentially contributing to prezygotic reproductive isolation. Particularly since all of our test genera potentially encounter each other in nature [53, 54], we further hypothesized that discrimination behavior among them based on CHC signaling will generally be enhanced to avoid fitness costs associated with heterospecific matings [55]. Accordingly, our behavioral assays were complemented with a comparative analysis between male and female CHC profiles to investigate whether they cluster in sex- and species-specific groups and whether the female CHC divergence reflects the differences in male CHC perception and species discrimination.

## Methods

### Experimental strains and rearing conditions

In the behavioral assays and for the chemical analyses we used laboratory strains from *Nasonia vitripennis*, *Trichomalopsis sarcophagae* and *Muscidifurax uniraptor*. For *N. vitripennis*, we used the strain ITA II, originally collected 2006 in Piedmont, Italy, and provided by L.W. Beukeboom, University of Groningen, The Netherlands. The *T. sarcophagae* strain was ordered from Beneficial Insectary Inc.^©^, Redding, Canada, provided by J. Gadau, Arizona State University, USA, and originally collected in 2004 in Alberta, Canada. The *M. uniraptor* strain originated from individuals collected in 1963 in Aibonito, Puerto Rico [48] and was provided by J.H. Werren, University of Rochester, USA. *M. uniraptor* constitutes a naturally parthenogenetic species [48, 49] that only tends to produce males under laboratory conditions [50], as was the case for our test strain.

All strains were kept in round plastic vials (97 mm height × 48 mm diameter) closed with foam plugs. In these plastic vials pupae of the blowfly *Calliphora erythrocephala* (MEIGEN) were provided as hosts. The strains were kept in an incubator (RUMED, Rubarth Apparate GMBH, Laatzen, Germany) under a constant temperature of 25 °C and a light/dark cycle of 16/8 h. These standardized conditions lead to a generation time of approximately 14 days for wasps from the genus *Nasonia* [56], whereas *T. sarcophagae* and *M. uniraptor* showed a generation time of approximately 16 days under these conditions. Wasps were collected from their blowfly hosts in the pupal stage before eclosure and their sex was determined according to the presence or absence of the ovipositor. Male and female pupae were subsequently kept separately in groups of about 20 individuals to ensure their virginity at the beginning of the mating trials. After eclosure, the adult wasps were allowed to mature for 48–72 h before they were freeze-killed or exposed to a potential mating partner.

### Behavioral assays

All behavioral observations were carried out in a round mating chamber with a diameter of 1 cm and a height of 0.5 cm as described by Ruther et al. [57]. Experiments were initiated by simultaneously placing a male and a female wasp into the mating chamber and directly closing the opening with a cover slide. Single observations were carried out for five minutes under a stereomicroscope in 10-fold magnification and under cold light. For establishing the function of female CHC profiles as mating cues for the males, we first assessed mate acceptance rates of male wasps from all three tested genera by pairing them with differentially treated conspecific females (see below) and recording whether they show both courtship behavior and/or actual copulation attempts. Pteromalid male courtship behavior usually constitutes of mounting a female wasp and displaying a stereotypic sequence of head nods, typically indicating readiness to mate [58–60]. In male *M. uniraptor*, the head nod sequence is largely replaced by antennal sweeps while mounting the female and this very comparable stereotypical behavior was recorded as courtship for this species instead [61, 62]. For copulations, the male usually stops his courtship display and backs up to inject his penis (aedeagus) into the female’s genital opening [60]. The presence or absence of both of these stereotypical and well-characterized behaviors was recorded in all tested combinations.

Males were tested with conspecific females that were either (i) untreated, (ii) freeze-killed, (iii) “cleared” of their CHC profile (i.e., freeze-killed females kept for 24 h in hexane, effectively removing the whole CHC profile with minute to non-detectable amounts remaining on the cuticle [43]), and (iv) “cleared” and reconstituted with conspecific female CHC extract. The CHC extracts were obtained by placing two freeze-killed females for 10 min in a closed 1.5 ml GC-vial with a conical insert containing 10 μl of hexane. Reconstitution of the CHC profile was achieved by applying 5 μl of the extract (containing one female equivalent) directly after the extraction to conspecific CHC-cleared females [43]. Pre-trials with fractionated cuticle extracts showed that the sexual signaling function is mediated through the non-polar CHC hexane fraction, and not through the polar fraction (separated as CH_2_Cl_2_ fraction) [43].

**Fig. 1:**
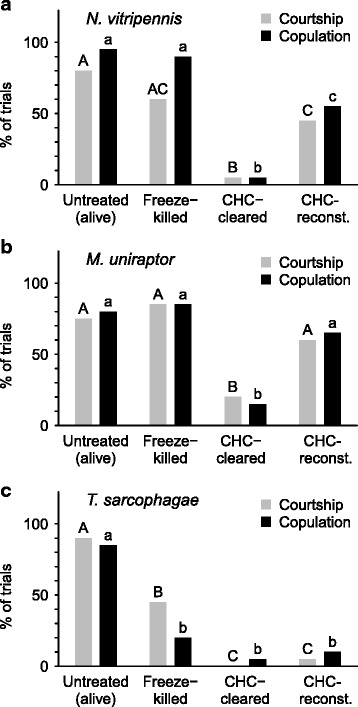
Percentages of male courtship (light grey bars) and copulation (black bars) with differentially treated conspecific freeze-killed females: **a**) *N. vitripennis*; **b**) *M. uniraptor*; **c**) *T. sarcophagae.* Left to right: Courtship behavior and copulation attempts with untreated (alive) conspecific females, freeze-killed conspecific females, freeze-killed conspecific females with cleared CHC profiles (‘CHC-cleared’), and freeze-killed conspecific females with reconstituted CHC profiles (‘CHC’-reconst.). 20 replicates performed for each treatment group, different letters indicate significant differences between treatment groups, upper-case letters are used for courtship behavior, lower-case letters for copulation attempts, compared independently by Benjamini-Hochberg corrected χ^2^ (Chi)-square tests, performed on absolute values

For establishing the species-specificity of female CHC signaling, we then proceeded to test male species discrimination by comparing courtship and copulatory behavior on con- vs. heterospecific freeze-killed females in all possible species combinations. Freeze-killed females were used as opposed to alive ones for two main reasons: First, using freeze-killed females in behavioral trials largely reduces other signaling modalities (e.g., behavioral, acoustic and tactile cues) and augments the importance of chemical cues for correctly identifying a potential mating partner [43, 63]. Second, completely removing or reconstituting CHC profiles has so far not been possible with alive Pteromalid females (Buellesbach & Schmitt, unpublished data). This is most probably due to the function of native CHC profiles as desiccation barrier [64, 65], rendering a complete removal or replacement without leaving traces of the original profile extremely difficult while simultaneously keeping the female alive.

**Fig. 2:**
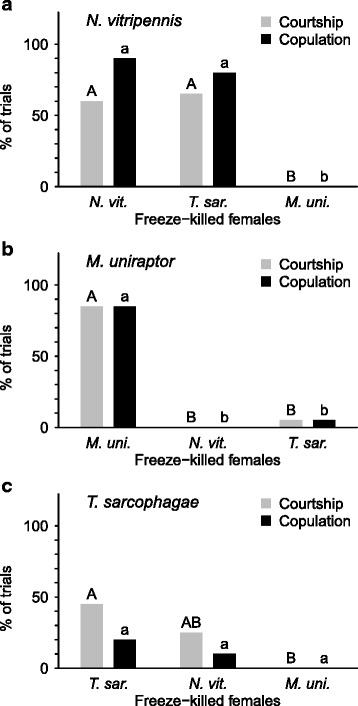
Percentages of male courtship (light grey bars) and copulation (black bars) with con- and heterospecific freeze-killed females: **a**) *N. vitripennis*; **b**) *M. uniraptor*; **c**) *T. sarcophagae*. Left: Courtship behavior and copulation attempts with conspecific freeze-killed females, respectively paired with the focal male species. Center and right: Courtship behavior and copulation attempts with heterospecific freeze-killed females. 20 replicates performed for each treatment group, different letters indicate significant differences between treatment groups, upper-case letters are used for courtship behavior, lower-case letters for copulation attempts, compared independently by Benjamini-Hochberg corrected χ^2^ (Chi)-square tests, performed on absolute values

To accommodate for their generally indifferent behavior towards freeze-killed females, we specifically designed additional assays for *T. sarcophagae* males. These assays were carried out like described above except that untreated (alive) con- and heterospecific females were used. In addition to male courtship behavior and copulation attempts, we also recorded the display of female receptivity in these assays, which is generally signaled by rising their abdomen and thus exposing their genital opening [60, 62].

**Fig. 3:**
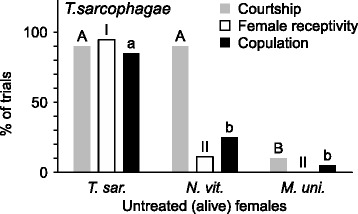
Percentages of male courtship (light grey bars) and copulation (black bars) paired with female receptivity (white bars) of *T. sarcophagae* males with untreated (alive) con- and heterospecific females: Left: Courtship behavior, triggered female receptivity and copulation attempts in pairings of *T. sarcophagae* males with untreated conspecific females. Center and right: Courtship behavior, triggered female receptivity and copulation attempts in pairings of *T. sarcophagae* males with untreated heterospecific *N. vitripennis* and *M. uniraptor* females, respectively. 20 replicates performed for each treatment group, different letters or numerals indicate significant differences between treatment groups, upper-case letters are used for courtship behavior, lower-case letters for copulation attempts, and roman numerals for female receptivity, compared independently by Benjamini-Hochberg corrected χ^2^ (Chi)-square tests, performed on absolute values

20 replicates were done per treatment, and the number of replicates where males showed courtship behavior or copulation attempts, or, in case of the assays with *T. sarcophagae* males and untreated (alive) females, where females signaled receptivity, were compared independently using χ^2^ (Chi)-square tests. If the χ^2^-square test was positive (*p* < 0.05), one-sided proportion tests were done as posthoc statistics. *P*-values from the posthoc tests were corrected for multiple testing by Benjamini-Hochberg corrections with the false discovery rate set to 0.05 [66]. All statistics were carried out with the program R and the package “stats” ([67], version 2.14.2).

### GC-MS analysis of the CHC profiles

Seventy nine male and female wasps of all three species were freeze-killed 48–72 h after eclosure and stored separately by sex and species in glass vials at − 20 °C. For CHC extraction, each wasp was placed for 10 min into 10 μl hexane. Extracts where then transferred to a fresh vial, concentrated by evaporating them with gaseous nitrogen to ~ 1 μl, and subsequently injected into a gas chromatograph coupled with a mass spectrometer (GC: 7890A; MS: 5975C; Agilent Technologies, Waldbronn, Germany) operating in electron impact ionisation mode at 70 eV and a source temperature of 230 °C. The entire sample was injected in a split/splitless injector in the splitless mode with an injector temperature of 250 °C. Separation of compounds was performed on a fused silica capillary column (HP-5 ms: 30 m; 0.25 mm^2^ ID; Agilent Technologies, Waldbronn, Germany) coated with a 0.25 μm (5%-phenyl)-methylpolysiloxane stationary phase with a temperature program starting from 60 °C and increasing by 40 °C per min to 200 °C, followed by an increase of 5 °C per min to 320 °C. Peak area integration and calculation as well as the identification of the substances were performed using the data analysis software Enhanced Chemstation G1701AA Version A.03.00 (Hewlett-Packard Company, Palo Alto, CA 94304 USA). CHC compounds were identified based on their retention indices and respective mass spectra, where possible [68] and expanded upon from previously reported CHC compositions in *Nasonia, Trichomalopsis* and *Muscidifurax* [42, 43, 53].

To make the Peak areas comparable between the species and sexes, to highlight the relative peak area differences and to correct for size-dependent variation, the peak areas were standardized by geometric means, based on the following formula:

$$ {S}_{i,j}=\frac{P_{i,j}}{\sqrt[n]{\prod_{j=1}^n{P}_j}} $$

with S_i,j_ referring to the standardized Peak area i of individual j, P_i,j_ to the absolute Peak area i of individual j and ^n^√ ∏ P_j_ to the geometric mean of all absolute Peak areas of individual j. Linear discriminant analysis (LDA) was performed utilizing the R package “MASS” [69] to test whether the differences in relative amounts of the CHC compounds sufficiently discriminate the six pre-defined groups into the three respective species and two sexes. To measure the quality of the LDA, Wilk’s λ, χ^2^ and the significance level of differentiation according to sex and species were calculated. To visualize the data by plotting the first three discriminant functions simultaneously, the R package “scatterplot3d” was used [70].

## Results

### Behavioral assays

In the pairings of *N. vitripennis* males with differentially treated conspecific females, both courtship behavior (CB: χ^2^ = 1.07, *P* = 0.18) and copulation attempts (CA: χ^2^ = 0, *P* = 0.5) were not significantly different between untreated (alive) and freeze-killed females (Fig. 1a).

However, when freeze-killed females were cleared of their CHC profiles, male courtship behavior and copulation attempts were significantly reduced compared to pairings with untreated (CB: χ^2^ = 20.05, *P* < 0.001; CA: χ^2^ = 28.9, *P* < 0.001) and freeze-killed females (CB: χ^2^ = 11.4, *P* = 0.001; CA: χ^2^ = 25.66, *P* < 0.001), respectively (Fig. 1a). Reconstituting the CHC profiles of previously CHC-cleared females resulted in a significant increase in male courtship behavior (CB: χ^2^ = 6.53, *P* = 0.011) and copulation attempts (CA: χ^2^ = 9.64, *P* = 0.002) compared to CHC-cleared females, restoring courtship to levels not significantly different from freeze-killed females (CB: χ^2^ = 0.4, *P* = 0.263; Fig. 1a). Male copulation attempts on CHC-reconstituted females, on the other hand, while showing a significant increase compared to CHC-cleared females (CA: χ^2^ = 9.64, *P* = 0.002), were still significantly lower than with untreated (CA: χ^2^ = 6.53, *P* = 0.008) and freeze-killed females (CA: χ^2^ = 4.51, *P* = 0.02; Fig. 1a).

Similarly, courtship behavior and copulation attempts from *M. uniraptor* males were not significantly different between untreated and freeze-killed females, respectively (CB: χ^2^ = 0.16, *P* = 0.654; CA: χ^2^ = 0, *P* = 0.5, Fig. 1b). Clearance of the CHC profiles resulted in significantly less courtship behavior and fewer copulation attempts than with both untreated (CB: χ^2^ = 10.03, *P* = 0.002; CA: χ^2^ = 14.44, *P* < 0.001) and freeze-killed females (CB: χ^2^ = 14.44, *P* < 0.001; CA: χ^2^ = 16.90, *P* < 0.001). Reconstituting the CHC profiles restored both tested behaviors to levels comparable with untreated (CB: χ^2^ = 0.46, *P* = 0.3; CA: χ^2^ = 0.5, *P* = 0.287) and freeze-killed females (CB: χ^2^ = 2.01, *P* = 0.118; CA: χ^2^ = 1.2, *P* = 0.205; Fig. 1b).

In contrast, *T. sarcophagae* males showed both significantly less courtship behavior and fewer copulation attempts with freeze-killed than with untreated females (CB: χ^2^ = 7.29, *P* = 0.005: CA: χ^2^ = 14.44, *P* < 0.001; Fig. 1c). More strikingly, no difference could be detected in both courtship behavior and copulation attempts between CHC-cleared and CHC-reconstituted females (CB: χ^2^ = 0, *P* = 0.5; CA: χ^2^ = 0, *P* = 0.5; Fig. 1c).

In con- vs. heterospecific comparisons (Fig. 2), there was no significant difference when *N. vitripennis* males were paired with conspecific and *T. sarcophagae* freeze-killed females, both in courtship behavior and copulation attempts (CB: χ^2^ = 0, *P* = 0.5; CA: χ^2^ = 0.2, *P* = 0.33, Fig. 2a). In contrast, freeze-killed *M. uniraptor* females were not courted and copulated with at all (CB: χ^2^ = 14.4, *P* < 0.001; CA: χ^2^ = 29.19, *P* < 0.001, Fig. 2a). An additional assay focusing on another *N. vitripennis* population collected in North America in close sympatry with other Pteromalid wasps resulted in a very similar pattern (Additional file 1).

*M. uniraptor* males both courted and copulated almost exclusively with conspecific freeze-killed females as compared to both heterospecific *N. vitripennis* (CB and CA: χ^2^ = 26.19, *P* < 0.001) and *T. sarcophagae* (CB and CA: χ^2^ = 22.73, *P* < 0.001) freeze-killed females.

In heterospecific pairings with *T. sarcophagae* males, courtship of freeze-killed *N. vitripennis* females was not significantly different from courtship of conspecifics (CB: χ^2^ = 0.99, *P* = 0.16), but significantly lower with freeze-killed *M. uniraptor* females that were not courted at all (CB: χ^2^ = 9.18, *P* = 0.004). No significant differences could be detected in copulation attempts of *T. sarcophaga*e males with con- and heterospecific freeze-killed females (CA: χ^2^ = 4.44, *P* = 0.108; Fig. 2c).

When paired with untreated (alive) females, *T. sarcophagae* males initially showed courtship behavior to the same extent with conspecific and *N. vitripennis* females (CB: χ^2^ = 0, *P* = 0.5). Female receptivity signaling, however, was significantly lower in the latter, heterospecific pairing (FR: χ^2^ = 21.85, *P* < 0.001), mirroring significantly fewer copulation attempts (CA: χ^2^ = 12.22, *P* < 0.001). Finally, compared to the conspecific pairing, untreated *M. uniraptor* females were significantly less courted (CB: χ^2^ = 22.5, *P* < 0.001), copulated with (CA: χ^2^ = 22.73, *P* < 0.001), and signaled no receptivity at all (FR: χ^2^ = 6.27, *P* = 0.009) when paired with *T. sarcophagae* males.

### Chemical analysis of the CHC profiles

**Fig. 4:**
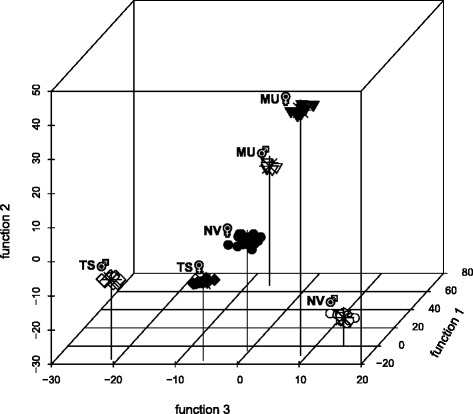
Three-dimensional linear discriminant analysis (LDA) simultaneously plotting the first three discriminant functions based on the CHC profile differences of the three tested wasp genera. NV = *N. vitripennis*, represented by circles, MU = *M. uniraptor,* represented by triangles and TS = *T. sarcophagae*, represented by diamonds. Males and females are represented by open and closed symbols, respectively, *n* = 79

A total of 79 individuals were pre-defined into six groups, constituting the three wasp genera with both respective sexes, for a linear discriminant analysis (LDA) according to their CHC profile differences (Fig. 4). All profiles were significantly differentiated according to sex and species (Wilk’s λ < 0.001, χ^2^ = 1199.36, *P* < 0.001). Discriminant function 1 accounts for 47.7%, function 2 for 30.4% and function 3 for 10.8% of the total variation, amounting to 88.9% of total variance explained by the first three functions. Plotted simultaneously, each group is uniquely defined, and no visual overlap between any of the six groups can be found (Fig. 4). Male CHC profiles cluster far apart from each other according to their respective species affiliation. *N. vitripennis* and *T. sarcophagae* female CHC profiles, however, cluster more closely together, whereas *M. uniraptor* female profiles cluster further away from both of them. A detailed description of the identified CHC compounds and relative CHC abundances per sex and species that form the basis for the divergent clusters is given in Additional file 2.

## Discussion

Our study aimed at elucidating the reliance on and species-specificity of female CHC profiles as sexual cues in three closely related parasitoid wasp genera. First, we investigated and compared both male courtship behavior and copulation attempts with conspecific untreated (alive) females and differentially treated conspecific freeze-killed females (Fig. 1). With freeze-killed females, other signaling modalities, e.g., behavioral, acoustic and tactile cues, are reduced and kept to a minimum, largely augmenting the reliance on chemical cues [43, 63]. Both *N. vitripennis* and *M. uniraptor* males displayed significantly less courtship and copulation attempts with freeze-killed females whose CHC profiles have been cleared than with freeze-killed females whose CHC profiles have been reconstituted (Fig. 1ab). This confirms female CHC as the main cues for sexual attraction when other signaling modalities potentially emitted from alive females are not present. However, under the same conditions, CHC are apparently not sufficient in *T. sarcophagae* for triggering male copulation behavior (Fig. 1c). Reconstitution of CHC profiles on freeze-killed females did not result in any more courtship or copulation attempts from males as compared to both CHC-cleared and untreated freeze-killed females. Thus, the central role of CHC as cue for female sexual attractiveness in absence of other potential cues emitted from alive females could not be confirmed here for *T. sarcophagae*. This opposes both our findings for *M. uniraptor* and *N. vitripennis* as well as other *Nasonia* species [43, 63]. Interestingly, in the phylogenetically most distant genus in this study represented by *M. uniraptor* [45], the female CHC function as sexual cues appears to be fully intact, a surprising finding as males only occur under laboratory conditions in this naturally parthenogenic species [48–50]. Our results suggest that additional cues, potentially emitted from alive females, are necessary for *T. sarcophagae* males for triggering their natural copulation behavior with the signaling of female receptivity appearing to be the most likely candidate cue (see Fig. 3).

Attempting to assess the species-specificity of this behavior, we also conducted male mate choice assays on con- vs. heterospecific freeze-killed females. Surprisingly, although clearly distinct and chemically different from each other (Fig. 4, Additional file 2), *N. vitripennis* males do not discriminate against heterospecific freeze-killed *T. sarcophagae* females but only against freeze-killed *M. uniraptor* females (Fig. 2a). As a cosmopolitan species documented to encounter *T. sarcophagae* [54] as well as other Pteromalid species in close sympatry [53, 71, 72], pronounced discrimination behavior would have been expected particularly in *N. vitripennis*. Although acceptance of heterospecific mates has been shown in *N. vitripennis* in behavioral assays with other *Nasonia* species [44, 63, 73], the extension of this non-selective behavior to a phylogenetically even more distant genus was surprising. Concerning population-specific variation, *N. vitripennis* CHC profiles have been found to be remarkably stable across populations irrespective of their geographic origin stemming from Europe or North America (Buellesbach, Diao, Beukeboom & Schmitt., unpublished data). Moreover, males from an *N. vitripennis* population collected in North America also showed non-discriminatory behavior against freeze-killed *T. sarcophagae* females (Additional file 1), suggesting that CHC profiles and associated behaviors constitute genetically fixed, species-specific traits with low intraspecific variation, which is also in accordance with studies on other *Nasonia* species [41, 43, 72].

Theory predicts that discriminatory behavior is generally enhanced in species potentially encountering closely related species in sympatry, especially if postzygotic hybridization barriers inflict fitness costs on interspecific reproduction [55, 74, 75]. In the *Nasonia* genus, postzygotic reproductive isolation mediated by different species-specific strains of *Wolbachia* bacteria leads to a substantial or total reduction of hybrid offspring in interspecific crosses [76–78]. Similarly, lab crosses between *N. vitripennis* and *T. sarcophagae* yielded substantially less hybrid offspring than conspecific control crosses (reduction by more than 60%, Additional file 3), hinting at considerable fitness costs inflicted on heterospecific courtship and copulations between these two species. Moreover, both are gregarious, i.e. multiple individual wasps can emerge from a single host pupa, and display both partially overlapping geographic ranges and shared host preferences [54], strongly suggesting an increased potential for sympatric encounters in nature. Apparently, female CHC profiles do not appear to be sufficiently distinguishable for *N. vitripennis* males to prevent interspecific courtship and copulations since they elicit both types of mating behaviors even with females from a different genus. These results contrast general assumptions that CHC can serve as sufficiently distinguishable discriminatory cues contributing to prezygotic reproductive isolation particularly on higher taxonomic levels [16, 79, 80].

In *T. sarcophagae* where female CHC do not even appear to sufficiently function as sexual cues when isolated from alive females, different signaling modalities potentially took precedence. Pairing *T. sarcophagae* males with untreated con- and heterospecific females while simultaneously recording female receptivity signaling interestingly revealed that males did not discriminate between conspecific and *N. vitripennis* females in their initial courtship behavior (Fig. 3). Copulation attempts and female receptivity signaling, however, both occurred significantly more often with con- than with heterospecific females. This clearly suggests that behavioral cues provided by alive females are essential for *T. sarcophagae* males to trigger natural copulation behavior, whereas courtship behavior appears to be similarly unspecific as in *N. vitripennis* males. The main behavioral trigger appears to be the signaling of female receptivity for the males to initiate copulation, which also has been found to be an additional important cue in the general mating behavior in *Nasonia* [59, 60] and other Pteromalid species [81, 82]. However, we cannot exclude the possibility that *T. sarcophagae* males possess the general capability to perceive female CHC as sexual cues. But, as opposed to our other tested genera and most other *Nasonia* species [43, 63], they do require additional cues and CHC are not sufficient when they are isolated.

Intriguingly, a similar inconsistency has been found in populations of the *Nasonia* species *N. giraulti*, where conspecific female CHC extracts also do not function as sexual cues when singled out [43, 44]. Taken the present findings for *T. sarcophagae* into account, *N. giraulti* males from certain populations potentially also require additional cues either not present in CHC extracts [44] or emitted from alive females for successful recognition and ultimately reproduction with conspecific females. These findings emphasize the potential importance of additional behavioral cues when studying chemical communication and also hint at the necessity for the presence and interaction of various communication modalities to trigger natural mating behavior. Our study suggests that the presence or absence of the reliance on certain communication cues to trigger mating behavior can, in fact, be a species-specific trait that may contribute to prezygotic isolation from other sympatric species and genera.

In *M. uniraptor*, female CHC apparently have retained their sexual signaling capability, being clearly perceivable and preferred by conspecific males (Figs. 1 and 2b), despite the fact that males are naturally absent in *M. uniraptor* populations. This contradicts the theory that sexual signaling characteristics largely disappear if species have become parthenogenetic over longer periods of time [49, 83]. Moreover, *M. uniraptor* are not only phylogenetically the most divergent [45], but also in terms of their CHC profiles (Fig. 4), potentially explaining that no courtship or copulations were recorded at all for *N. vitripennis* (Fig. 2a) and *T. sarcophagae* (Fig. 2b) males with freeze-killed *M. uniraptor* females.

## Conclusions

We found remarkable differences in the reliance on CHC as both sexual stimulant and species-specific recognition cues in our tested parasitoid wasp genera. The function as female sexual cues in isolation of other stimuli could not be confirmed as a universal CHC characteristic, nor did CHC mediate distinctive enough recognition behavior to reliably function as prezygotic hybridization barrier between all tested genera. This strongly suggests that in order to obtain a holistic view on sexual signaling in relation to reproductive isolation, single signaling modalities such as CHC should be regarded in conjunction with other communication modes rather than isolated [11, 84]. In larger phylogenetic contexts, several different signaling modalities potentially comprise a rather complex, synergistic network ensuring the functionality of species-specific sexual communication, though this has rarely been assessed empirically so far [21, 22, 85, 86].

Future studies should direct more effort into incorporating and integrating these different communication modalities, presumably complementing each other to unfold their full potential as prezygotic hybridization barriers. Overall, interactions of various communication modalities and differences in the reliance on each one of them might be an important factor contributing to the divergence of populations, prezygotic reproductive isolation and, ultimately, speciation [55, 75, 87].

## Additional files

Additional file 1: Percentages of male courtship (light grey bars) and copulation (black bars) with con- and heterospecific freeze-killed females tested with males and females from an *N. vitripennis* population originally collected in 2006 in New York, North America (N**.**A**.**). 20 replicates performed for each treatment group, different letters indicate significant differences between treatment groups, upper-case letters are used for courtship behavior, lower-case letters for copulation attempts, compared independently by Benjamini-Hochberg corrected χ^2^ (Chi)-square tests, performed on absolute values. (PDF 52 kb)

Additional file 2: CHC compounds identified from males and females of *Nasonia vitripennis*, *Trichomalopsis sarcophagae,* and *Muscidifurax uniraptor*. Compound identifications, retention indices (RI), and their mean relative abundances (%) as well as standard deviations (± %) for each respective sex and species are given. X indicates non-detectable amounts of the respective compounds. (DOCX 30 kb)

Additional file 3: F1 male and female offspring from crosses between *T. sarcophagae* males with conspecific virgin *T. sarcophagae* and heterospecific *N. vitripennis* females, respectively. Note that only female offspring constitutes hybrid offspring as Hymenopteran males develop from haploid, unfertilized eggs. The *N. vitripennis* strain used for the crosses has been antibiotically cured of its *Wolbachia* infection and was originally collected in Leiden, The Netherlands. (DOCX 13 kb)
