# Supplementary figures and images for: Differences in the reliance on cuticular hydrocarbons as sexual signaling and species discrimination cues in parasitoid wasps
Source: Front Zool. 2018 May 10;15:22. doi: 10.1186/s12983-018-0263-z (PMC5946414; doi:10.1186/s12983-018-0263-z)

*N. vitripennis* (N.A.)

■ Courtship  
■ Copulation

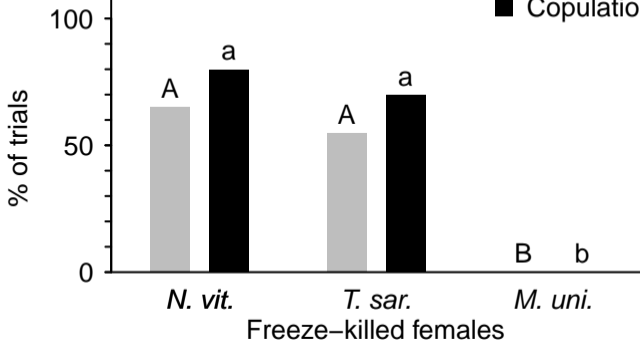

Supplement: Supplementary file 1 — Percentages of male courtship (light grey bars) and copulation (black bars) with con- and heterospecific freeze-killed females tested with males and females from an N. vitripennis population originally collected in 2006 in New York, North America (N.A.). 20 replicates performed for each treatment group, different letters indicate significant differences between treatment groups, upper-case letters are used for courtship behavior, lower-case letters for copulation attempts, compared independently by Benjamini-Hochberg corrected χ2 (Chi)-square tests, performed on absolute values. (PDF 52 kb) [file 12983_2018_263_MOESM1_ESM.pdf]
