# Supplementary material for: Differences in the reliance on cuticular hydrocarbons as sexual signaling and species discrimination cues in parasitoid wasps
Source: Front Zool. 2018 May 10;15:22. doi: 10.1186/s12983-018-0263-z (PMC5946414; doi:10.1186/s12983-018-0263-z)
Supplement: Supplementary file 2 — CHC compounds identified from males and females of Nasonia vitripennis, Trichomalopsis sarcophagae, and Muscidifurax uniraptor. Compound identifications, retention indices (RI), and their mean relative abundances (%) as well as standard deviations (± %) for each respective sex and species are given. X indicates non-detectable amounts of the respective compounds. (DOCX 30 kb) [file 12983_2018_263_MOESM2_ESM.docx]

**Additional file 2:** CHC compounds identified from males and females of *Nasonia* *vitripennis*. *Trichomalopsis sarcophagae* and *Muscidifurax uniraptor* with their retention indices, respective mean quantities and standard deviations in percent. An X indicates non-detectable amounts of the respective compound.

|  |  | *N. vitripennis* | | *T. sarcophagae* | | *M. uniraptor* | |
| --- | --- | --- | --- | --- | --- | --- | --- |
| CHC identification | RI | ♀ | ♂ | ♀ | ♂ | ♀ | ♂ |
|  |  |  |  |  |  |  |  |
| C29 | 2897 | 7.21 | 10.32 | 5.86 | 6.66 | 7.36 | 16.36 |
|  |  | ± 1.77 | ± 7.05 | ± 1.12 | ± 3.27 | ± 5.88 | ± 6.66 |
| 9-; 11-; 13-; 15-MeC29 | 2928 | 1.13 | 0.53 | 1.24 | 1.41 | 0.17 | 0.16 |
|  |  | ± 0.79 | ± 0.48 | ± 0.33 | ± 0.69 | ± 0.07 | ± 0.09 |
| 7-MeC29 | 2938 | 3.4 | 2.73 | 1.95 | 5.71 | 0.23 | 0.33 |
|  |  | ± 1.82 | ± 1.33 | ± 0.24 | ± 1.53 | ± 0.09 | ± 0.17 |
| 5-MeC29 | 2948 | 0.98 | 1.1 | 0.73 | 1.97 | 0.06 | 0.1 |
|  |  | ± 0.51 | ± 0.56 | ± 0.13 | ± 0.62 | ± 0.02 | ± 0.08 |
| 3-MeC29 | 2971 | 0.28 | 0.29 | 1.48 | 0.66 | 1.88 | 1.23 |
|  |  | ± 0.16 | ± 0.20 | ± 0.33 | ± 0.32 | ± 1.66 | ± 0.64 |
| 15,17-DiMeC29 | 2976 | 0.31 | 0.43 | 0.26 | 0.38 | 0.08 | 0.07 |
|  |  | ± 0.27 | ± 0.25 | ± 0.08 | ± 0.08 | ± 0.04 | ± 0.03 |
| C30 | 2996 | 1.45 | 0.97 | 1.19 | 0.81 | 1.12 | 1.59 |
|  |  | ± 0.30 | ± 0.27 | ± 0.17 | ± 0.19 | ± 0.71 | ± 0.31 |
| 3,7-DiMeC29 | 3003 | 0.25 | 0.19 | 0.28 | 0.41 | 1.22 | 0.12 |
|  |  | ± 0.19 | ± 0.11 | ± 0.04 | ± 0.19 | ± 0.82 | ± 0.07 |
| 3,11,15-TriMeC29 | 3028 | 0.20 | 0.05 | 0.19 | 0.09 | 0.04 | 0.07 |
|  |  | ± 0.12 | ± 0.02 | ± 0.05 | ± 0.03 | ± 0.01 | ± 0.02 |
| 7-MeC30 | 3035 | 0.53 | 0.6 | 0.75 | 1.45 | 0.19 | 0.09 |
|  |  | ± 0.23 | ± 0.14 | ± 0.11 | ± 0.21 | ± 0.13 | ± 0.03 |
| 5-MeC30 | 3046 | 0.05 | 0.08 | 0.1 | 0.15 | 0.00 | 0.01 |
|  |  | ± 0.03 | ± 0.03 | ± 0.02 | ± 0.01 | ± 0.01 | ± 0.00 |
| 4-MeC30 | 3055 | 0.16 | 0.13 | 0.21 | 0.14 | 0.24 | 0.07 |
|  |  | ± 0.08 | ± 0.04 | ± 0.06 | ± 0.04 | ± 0.13 | ± 0.02 |
| 3-MeC30 | 3071 | 0.11 | X | 0.21 | 0.08 | 0.12 | 0.08 |
|  |  | ± 0.05 |  | ± 0.02 | ± 0.03 | ± 0.07 | ± 0.03 |
| 7-C31ene | 3076 | 0.13 | 1.64 | 0.07 | 0.17 | 0.04 | X |
|  |  | ± 0.11 | ± 0.93 | ± 0.04 | ± 0.07 | ± 0.06 |  |
| 9-C31ene | 3083 | 0.08 | 1.29 | 0.06 | 0.12 | 0.37 | X |
|  |  | ± 0.06 | ± 0.60 | ± 0.03 | ± 0.05 | ± 0.15 |  |
| 4,8- oder 4,22-DiMeC30 | 3090 | X | X | X | X | 16.33 | X |
|  |  |  |  |  |  | ± 2.91 |  |
| C31 | 3097 | 21.06 | 17.19 | 13.31 | 8.65 | X | 16.60 |
|  |  | ± 5.26 | ± 4.37 | ± 2.58 | ± 2.39 |  | ± 2.32 |
|  |  | *N. vitripennis* | | *T. sarcophagae* | | *M. uniraptor* | |
| CHC identification | RI | ♀ | ♂ | ♀ | ♂ | ♀ | ♂ |
| 9-; 11-; 13-; 15-MeC31 | 3129 | 7.99 | 1.98 | 9.64 | 4.67 | 1.25 | 2.86 |
|  |  | ± 0.82 | ± 0.41 | ± 0.72 | ± 0.54 | ± 0.25 | ± 0.89 |
| 7-MeC31 | 3138 | 11.52 | 18.89 | 11.17 | 27.01 | 1.36 | 2.18 |
|  |  | ± 1.58 | ± 5.01 | ± 1.17 | ± 2.61 | ± 0.31 | ± 0.65 |
| 5-MeC31 | 3147 | 6.54 | 5.98 | 5.5 | 7.83 | 1.09 | 0.93 |
|  |  | ± 0.44 | ± 1.63 | ± 0.31 | ± 0.70 | ± 0.20 | ± 0.29 |
| 9,21-; 15,11-DiMeC31 | 3158 | 1.07 | 0.42 | 1.14 | 0.83 | 0.29 | 0.23 |
|  |  | ± 0.15 | ± 0.13 | ± 0.15 | ± 0.14 | ± 0.05 | ± 0.09 |
| 7,11-; 7,23-DiMeC31 | 3165 | 1.28 | 1.55 | 3.07 | 2.23 | 0.82 | 0.59 |
|  |  | ± 0.31 | ± 0.41 | ± 0.27 | ± 0.81 | ± 0.24 | ± 0.33 |
| 3-MeC31 | 3172 | 5.65 | 4.41 | 6.98 | 4.84 | 7.22 | 9.1 |
|  |  | ± 2.08 | ± 1.46 | ± 1.39 | ± 1.92 | ± 3.31 | ± 2.26 |
| 5,9-; 5,25-DiMeC31 | 3177 | 1.8 | 3.69 | 2.33 | 3.77 | 0.88 | 0.3 |
|  |  | ± 0.80 | ± 1.40 | ± 0.31 | ± 1.63 | ± 0.19 | ± 0.11 |
| 7,9-DiMeC31 | 3186 | 0.62 | 0.95 | 0.59 | 1.31 | 0.28 | 0.21 |
|  |  | ± 0.22 | ± 0.42 | ± 0.10 | ± 0.59 | ± 0.04 | ± 0.07 |
| 7,21-; 3,15-DiMeC31 | 3194 | 1.19 | 0.82 | 1.33 | 0.77 | 0.67 | 0.81 |
|  |  | ± 0.15 | ± 0.12 | ± 0.22 | ± 0.08 | ± 0.12 | ± 0.18 |
| 3,7-DiMeC31 | 3205 | 0.67 | 1.28 | 0.94 | 0.93 | 7.89 | 0.22 |
|  |  | ± 0.15 | ± 0.45 | ± 0.05 | ± 0.21 | ± 1.07 | ± 0.10 |
| 7-; 8-MeC31 | 3231 | 0.84 | 0.57 | 1.31 | 0.87 | 1.61 | 0.36 |
|  |  | ± 0.16 | ± 0.20 | ± 0.13 | ± 0.12 | ± 0.54 | ± 0.14 |
| 5-; 6-MeC31 | 3242 | 0.23 | 0.46 | 0.22 | 0.45 | 0.35 | 0.07 |
|  |  | ± 0.06 | ± 0.24 | ± 0.03 | ± 0.12 | ± 0.18 | ± 0.03 |
| 4-MeC32 | 3255 | 0.74 | 0.14 | 1.48 | 0.83 | 0.52 | 0.22 |
|  |  | ± 0.19 | ± 0.06 | ± 0.55 | ± 0.23 | ± 0.04 | ± 0.08 |
| 7-C33ene | 3276 | 0.06 | 0.31 | 0.23 | 0.15 | 0.08 | 0.05 |
|  |  | ± 0.03 | ± 0.16 | ± 0.16 | ± 0.08 | ± 0.04 | ± 0.02 |
| 9-C33ene | 3285 | 0.12 | 0.96 | 0.2 | 0.13 | 1.03 | 0.04 |
|  |  | ± 0.05 | ± 0.45 | ± 0.07 | ± 0.05 | ± 0.18 | ± 0.04 |
| C33 | 3297 | 0.38 | 0.71 | 0.29 | 0.17 | 2.17 | 0.74 |
|  |  | ± 0.13 | ± 0.20 | ± 0.07 | ± 0.05 | ± 0.69 | ± 0.29 |
| 9-; 11-; 13-; 15-MeC33 | 3327 | 4.32 | 1.54 | 6.98 | 3.22 | 1.39 | 5.81 |
|  |  | ± 0.60 | ± 0.45 | ± 0.70 | ± 0.53 | ± 0.41 | ± 2.91 |
| 7-MeC33 | 3334 | 0.95 | 1.37 | 0.84 | 0.99 | 0.27 | 0.65 |
|  |  | ± 0.17 | ± 0.49 | ± 0.13 | ± 0.13 | ± 0.06 | ± 0.34 |
| 5-MeC33 | 3347 | 2.71 | 0.66 | 3.64 | 1.74 | 1.16 | 0.25 |
|  |  | ± 0.57 | ± 0.22 | ± 0.75 | ± 0.38 | ± 0.27 | ± 0.08 |
|  |  |  |  |  |  |  |  |
|  |  | *N. vitripennis* | | *T. sarcophagae* | | *M. uniraptor* | |
| CHC identification | RI | ♀ | ♂ | ♀ | ♂ | ♀ | ♂ |
|  |  |  |  |  |  |  |  |
| 11,15-; 11,21-DiMeC33 | 3359 | 2.63 | 0.58 | 2.03 | 0.73 | 0.86 | 10.18 |
|  |  | ± 0.39 | ± 0.21 | ± 0.21 | ± 0.20 | ± 0.23 | ± 1.34 |
| 7,19-; 7,23-DiMeC33 | 3364 | 1.96 | 4.01 | 1.14 | 1.88 | 3.16 | 3.53 |
|  |  | ± 0.27 | ± 1.32 | ± 0.29 | ± 1.12 | ± 0.62 | ± 0.83 |
| 5,9-DiMeC33 | 3376 | 3.04 | 3.68 | ± 2.6 | 2.07 | 0.59 | ± 1.9 |
|  |  | ± 0.41 | ± 1.05 | 0.91 | ± 0.90 | ± 0.15 | 0.47 |
| 3,15-; 3,17-DiMeC33 | 3399 | 0.66 | 0.59 | ± 0.81 | 0.29 | 0.35 | 0.33 |
|  |  | ± 0.16 | ± 0.26 | 1.20 | ± 0.13 | ± 0.09 | ± 0.13 |
| 5,9,21-; 5,9,13-TriMeC33 | 3405 | 0.27 | 1.26 | ± 0.82 | 0.23 | 4.65 | 0.24 |
|  |  | ± 0.11 | ± 0.60 | 0.48 | ± 0.18 | ± 1.56 | ± 0.06 |
| 3,7,11-TriMeC33 | 3426 | 0.16 | 0.16 | ± 0.26 | 0.12 | 0.41 | 0.32 |
|  |  | ± 0.06 | ± 0.06 | 0.11 | ± 0.06 | ± 0.16 | ± 0.09 |
| 8,10-; 8,12-; 8,14-; 8,16-; | 3434 | 0.16 | 0.37 | ± 0.21 | 0.09 | 5.62 | 0.14 |
| 8,18-DiMeC34 |  | ± 0.08 | ± 0.18 | 0.10 | ± 0.06 | ± 1.14 | ± 0.05 |
| 3,7,11,15-TetraMeC33 | 3450 | 0.29 | 0.20 | ± 0.71 | 0.24 | 1.06 | 0.62 |
|  |  | ± 0.12 | ± 0.16 | ± 0.33 | ± 0.14 | ± 0.36 | ± 0.16 |
| 11-; 13-; 15-; 17-MeC35 | 3525 | 0.53 | 0.36 | 0.72 | 0.35 | 0.37 | 2.37 |
|  |  | ± 0.18 | ± 0.15 | ± 0.32 | ± 0.18 | ± 0.17 | ± 0.67 |
| 15,19-; 13,17-; 11,15-DiMeC35 | 3550 | 1.52 | 0.3 | 2.39 | 0.74 | 4.74 | 8.27 |
|  |  | ± 0.37 | ± 0.15 | ± 0.71 | ± 0.43 | ± 1.86 | ± 2.22 |
| 7,15-;7,19-;7,23-DiMeC35 | 3564 | 0.47 | 1.39 | 0.35 | 0.39 | 0.38 | 0.9 |
|  |  | ± 0.17 | ± 0.61 | ± 0.22 | ± 0.28 | ± 0.14 | ± 0.25 |
| 5,17-DiMeC35 | 3572 | 1.01 | 1.48 | 0.99 | 0.55 | 2.25 | 5.11 |
|  |  | ± 0.34 | ± 0.61 | ± 0.45 | ± 0.30 | ± 0.91 | ± 2.34 |
| 3,15-DiMeC35 | 3599 | 0.21 | 0.35 | 0.19 | 0.1 | 0.72 | 0.92 |
|  |  | ± 0.12 | ± 0.18 | ± 0.12 | ± 0.06 | ± 0.26 | ± 0.48 |
| 3,7-DiMeC35 | 3607 | X | X | X | X | 0.54 | X |
|  |  |  |  |  |  | ± 0.25 |  |
| 3,7,19-TriMeC35 | 3630 | X | X | X | X | 8.98 | X |
|  |  |  |  |  |  | ± 3.35 |  |
| 15,17-DiMeC36 | 3647 | X | X | X | X | 1.56 | X |
|  |  |  |  |  |  | ± 0.61 |  |
| unknown | 3653 | 0.07 | 0.05 | 0.11 | 0.05 | X | 0.58 |
|  |  | ± 0.04 | ± 0.02 | ± 0.08 | ± 0.02 |  | ± 0.45 |
| 15-; 17-; 19-MeC37 | 3723 | 0.15 | 0.13 | 0.09 | 0.07 | 0.12 | 0.16 |
|  |  | ± 0.10 | ± 0.08 | ± 0.05 | ± 0.04 | ± 0.07 | ± 0.05 |
| 11,25-; 11,27-DiMeC37 | 3747 | 0.36 | 0.16 | 0.39 | 0.16 | 1.94 | 0.6 |
|  |  | ± 0.16 | ± 0.09 | ± 0.16 | ± 0.06 | ± 0.85 | ± 0.19 |
|  |  |  | |  | |  | |
|  |  |  | |  | |  | |
|  |  |  | |  | |  | |
|  |  | *N. vitripennis* | | *T. sarcophagae* | | *M. uniraptor* | |
| CHC identification | RI | ♀ | ♂ | ♀ | ♂ | ♀ | ♂ |
|  |  |  |  |  |  |  |  |
| 7,21-DiMeC37 | 3761 | 0.13 | 0.32 | 0.09 | 0.11 | 0.4 | 0.11 |
|  |  | ± 0.08 | ± 0.10 | ± 0.06 | ± 0.03 | ± 0.18 | ± 0.06 |
| 5,15-; 5,17-DiMeC37 | 3771 | 0.21 | 0.24 | 0.2 | 0.12 | 1.16 | 0.56 |
|  |  | ± 0.12 | ± 0.08 | ± 0.11 | ± 0.04 | ± 0.55 | ± 0.30 |
