# Supplementary material for: Differences in the reliance on cuticular hydrocarbons as sexual signaling and species discrimination cues in parasitoid wasps
Source: Front Zool. 2018 May 10;15:22. doi: 10.1186/s12983-018-0263-z (PMC5946414; doi:10.1186/s12983-018-0263-z)
Supplement: Supplementary file 3 — F1 male and female offspring from crosses between T. sarcophagae males with conspecific virgin T. sarcophagae and heterospecific N. vitripennis females, respectively. Note that only female offspring constitutes hybrid offspring as Hymenopteran males develop from haploid, unfertilized eggs. The N. vitripennis strain used for the crosses has been antibiotically cured of its Wolbachia infection and was originally collected in Leiden, The Netherlands. (DOCX 13 kb) [file 12983_2018_263_MOESM3_ESM.docx]

**Additional file 3:** F1 male and female offspring from crosses between *T. sarcophagae* females with conspecific *T. sarcophagae* and heterospecific *N. vitripennis* males. The *N. vitripennis* strain used for the crosses has been antibiotically cured of its *Wolbachia* infection and was originally collected in Leiden, the Netherlands.

| Crossing scheme | F1 ♂ | F1 ♀ | Sex ratio ♀ / ♂ |
| --- | --- | --- | --- |
| *T. sar.* ♀ + *T. sar.* ♂ | 106 | 117 | 1.1 |
| *T. sar.* ♀ + *N. vit.* ♂ | 105 | 45 | 0.42 |
